# Ten Years Later: Molecular Detection and Circulation of Enteric Viruses in Italian Pig Herds

**DOI:** 10.1155/tbed/4011873

**Published:** 2026-10-30

**Authors:** Ilaria Di Bartolo, Fabio Ostanello, Giovanni Ianiro, Luca De Sabato, Chiara Settanni, Irene Berselli, David Ranucci, Marina Monini

**Affiliations:** ^1^ Department of Food Safety Nutrition and Veterinary Public Health Istituto Superiore di Sanità Viale Regina Elena, 299 Rome 00161 Italy iss.it; ^2^ Department of Veterinary Medical Sciences University of Bologna Via Tolara di Sopra 50, Ozzano dell’Emilia Bologna 40064 Italy unibo.it; ^3^ Department of Veterinary Medicine University of Perugia Via San Costanzo 4 Perugia 06121 Italy unipg.it

**Keywords:** asymptomatic infection, hepatitis E virus genotype 3, porcine astrovirus, porcine kobuvirus, porcine rotavirus A, swine enteric virome, viral infection

## Abstract

Enteric viral infections remain a major challenge for the swine industry, particularly in young animals, and represent a potential source of zoonotic risk. Ten years after a previous survey conducted in Italy, we performed a cross‐sectional study investigating mostly the same viruses to evaluate the persistence and circulation of different enteric viruses in Italian pig farms. A total of 240 pooled swine fecal samples from asymptomatic swine, collected from 12 pig farms in Northern and Central Italy, were analyzed for hepatitis E virus (HEV), porcine astrovirus (PAstV), porcine kobuvirus (PKoV), rotavirus A (RVA), caliciviruses (norovirus; NoV and sapovirus; SaV), and coronaviruses (CoVs). Overall, frequent circulation of enteric viruses was confirmed, with common codetection of multiple viruses within the same samples. PAstV and PKoV were each detected in 10 of 12 farms, followed by RVA in 9 farms, HEV in 6 farms, and caliciviruses in 1 farm; no CoV RNA was detected. PAstV, PKoV, HEV, RVA, and calicivirus RNA were detected in 70.0%, 51.2%, 27.1%, 19.6%, and 1.3% of pooled fecal samples, respectively, and 61.7% of pools contained RNA from two or more viruses. Caliciviruses were rarely detected, while no CoVs were identified, indicating either a limited circulation in the investigated farms or in fecal samples, being mostly associated with respiratory diseases. Associations between viral detection and pig age, supply‐chain affiliation, and herd size were assessed using generalized estimating equation (GEE) models accounting for within‐farm clustering. Although crude pool‐level detection frequencies differed among categories, no statistically significant associations were identified. Although comparison with previous studies should be interpreted cautiously because the sampled farms and some diagnostic assays were not identical, this study demonstrates the continued circulation of several enteric viruses in Italian pig farms. The result highlights the importance of molecular surveillance to safeguard animal health and assess risks of interspecies transmission.

## 1. Introduction

Pork meat is one of the most consumed animal products globally, making pig farming a key component for the security of pig‐derived food production systems.

To meet growing consumer demand, producers have intensified swine production systems, leading to higher animal farming densities and faster turnover rates. These factors contribute to increasing the susceptibility to disease outbreaks, causing a rise in both swine‐specific and zoonotic pathogens within the farms [1].

Several studies show that astroviruses (AstVs), kobuviruses (KoVs), and hepatitis E virus (HEV) are widespread in European pig farms [2–4]. While long‐term trend data remain limited, evidence suggests that these viruses are now widespread, posing persistent risks to both animals and, in the case of HEV genotype 3, potential relevance to public health due to its zoonotic potential.

Many viruses are frequently found, that is, HEV, in healthy animals, indicating subclinical endemic infections.

Furthermore, viral infections are symptomatic and can cause severe diarrhea and mortality in animals, especially in piglets, with economic losses to pig farmers, the spread of infections, and higher risk for humans when zoonotic pathogens are involved. A very broad spectrum of viruses that can cause porcine diarrhea has been found in pigs, including porcine sapelovirus (PSV), KoV, sapovirus (SaV), AstV, and porcine rotavirus [5]. More recently, novel technologies of sequencing allowed the discovery of novel viruses, some of which without clear clinical implication (e.g., torque teno virus, TTV) [6]. Moreover, coinfections are common, maybe causing a possible worsening of symptoms and potentially facilitating viral recombination or mutation [2–4]. Among enteric viruses, porcine AstV (PAstV) [7], SaV [8], and porcine KoV (PKoV) [9] have been detected in pig feces and are linked to clinical symptoms in neonatal pigs [10] but may not be associated with specific symptoms in older animals.

AstVs are nonenveloped RNA viruses infecting birds and mammals [11]. PAstV affects pigs, and the infection can be symptomatic, causing diarrhea and, in some cases, neurological disorders [12]. AstVs show high genetic diversity with frequent recombination events [13]. Some strains in pigs are related to human AstVs, as shown by phylogenetic analyses [14]. PAstVs cluster with human lineages and share conserved polymerase and capsid gene regions [14–16], suggesting a shared evolutionary ancestor, highlighting potential zoonotic risks and the need for ongoing monitoring [14, 15, 17].

Noroviruses (NoVs; family Caliciviridae) are recognized as the leading cause of acute gastroenteritis in humans and infect various animal species, including swine [18]. NoV strains infecting both pigs and humans belong to the same genogroup, GII [19, 20]. However, there is no evidence that porcine NoVs can infect humans. Sporadic detection of human genotypes in swine raises concerns about the possible role of pigs as reservoirs for strains infecting humans, but to date, no evidence of transmission has been reported [21]. In Europe, studies have identified porcine NoVs, particularly genotypes GII.11, GII.18, and GII.19, as being associated or not associated with the occurrence of diarrhea [19, 20, 22, 23]. However, the occurrence of nonspecific symptoms and the frequent coinfection of NoV with other pathogens make it difficult to clearly associate NoV with the occurrence of symptoms [18].

SaVs (family Caliciviridae) belong to the same family of NoVs and are detected in pig herds worldwide in all age groups but appear to be most prevalent in postweaning animals [24, 25]. These viruses possess a highly heterogeneous RNA genome and can infect several animal species. In swine, the most commonly identified strains belong to genogroups GIII and GVII [26]. Although frequently detected in healthy pigs, their role in piglet diarrhea is still unclear [27]. In Czech farms, SaV detection prevalence reached 28.3%, with a higher detection rate in postweaning pigs than in finisher pigs (9.8%) and nursing piglets (3.0%), while sows were negative, likely due to maternal antibodies [25, 28]. Despite genetic similarities to human strains, evidence for zoonotic transmission remains limited [25].

Group A rotavirus (rotavirus A [RVA], family Reoviridae) causes severe diarrhea in young animals and children [29]. Recent studies in Europe show high genetic diversity of porcine RVA strains [30]. During 2018–2021, a prevalence of about 50% was reported in domestic pigs and wild boars with multiple genotypes and evidence of interspecies transmission [31].

Hepatitis E virus (HEV, family Hepeviridae, genus *Paslahepevirus*) is a positive‐sense RNA virus causing acute hepatitis in humans [32]. Genotypes 3 and 4 (HEV‐3 and ‐4) are zoonotic and infect both humans and animals, particularly pigs and wild boars [33]. Weaner pigs are highly susceptible to zoonotic HEV, which is widespread in pig farms worldwide [34]. Being foodborne transmitted to humans, the whole food chain can be at risk of being HEV contaminated [35], especially at the farm level. A recent work identified rat HEV (species *Rocahepevirus ratti*) in a farmed pig population, which suggests that pigs might also act as carriers or amplifiers of uncommon HEV species [36].

Coronaviruses (CoVs) infect many mammals and birds, causing respiratory, digestive, hepatic, and nervous disorders [37]. Belonging to the Coronaviridae family, they are divided into four genera: *Alpha*‐, *Beta*‐, *Gamma*‐, and *Deltacoronavirus* [37, 38]. Their single‐stranded positive‐sense enveloped RNA encodes nonstructural and structural proteins [38].

To date, six CoVs have been found to infect pigs: four alpha‐CoVs (TGEV, PEDV, SADS‐CoV, and PRCV), one beta‐CoV (PHEV), and one delta‐CoV (PDCoV) [39–41]. Among these, TGEV, PEDV, PDCoV, and SADS‐CoV are responsible for enteric diseases marked by acute diarrhea, linked with mortality in newborn piglets and occasionally in sows [41, 42].

This study aimed to provide an updated assessment of the molecular detection and circulation of selected enteric viruses in pig farms in Northern and Central Italy, areas also investigated approximately 10 years earlier, and to descriptively compare the current findings with those of the previous survey [43]. The viral panel was also expanded to include PKoV and CoVs. The study also aimed to evaluate whether viral detection was associated with pig age and selected farm characteristics while accounting for the clustered sampling design.

## 2. Materials and Methods

### 2.1. Sample and Farm Description

From 2019 to 2025, a total of 240 pooled fecal stool samples were collected from 12 herds located in different Italian provinces. The 12 farms included 10 fattening farms, one farrow‐to‐finish farm, and one farrow‐to‐weaning farm. Six farms were located in Northern Italy (Lombardy and Emilia‐Romagna regions) and six in Central Italy (Lazio and Umbria regions) (Figure 1). Except for the five farms sampled in 2025, the other seven farms had already been investigated during a previous study, aiming to detect HEV [35]. The corresponding HEV detection results and sequence data are also included in the present study. The number and type of fecal samples to be examined were established in the previous study [35] in the framework of the One Health European Joint Programme, BIOPIGEE project, and were also applied for the most recently investigated farms. As reported in Ianiro et al. [35], the number of pooled samples was determined to estimate HEV prevalence under the assumptions of a perfect test (100% sensitivity and specificity), an expected true prevalence of 10%, a desired precision of ±5.5%, and a 95% confidence interval (95% CI).

**Figure 1:**
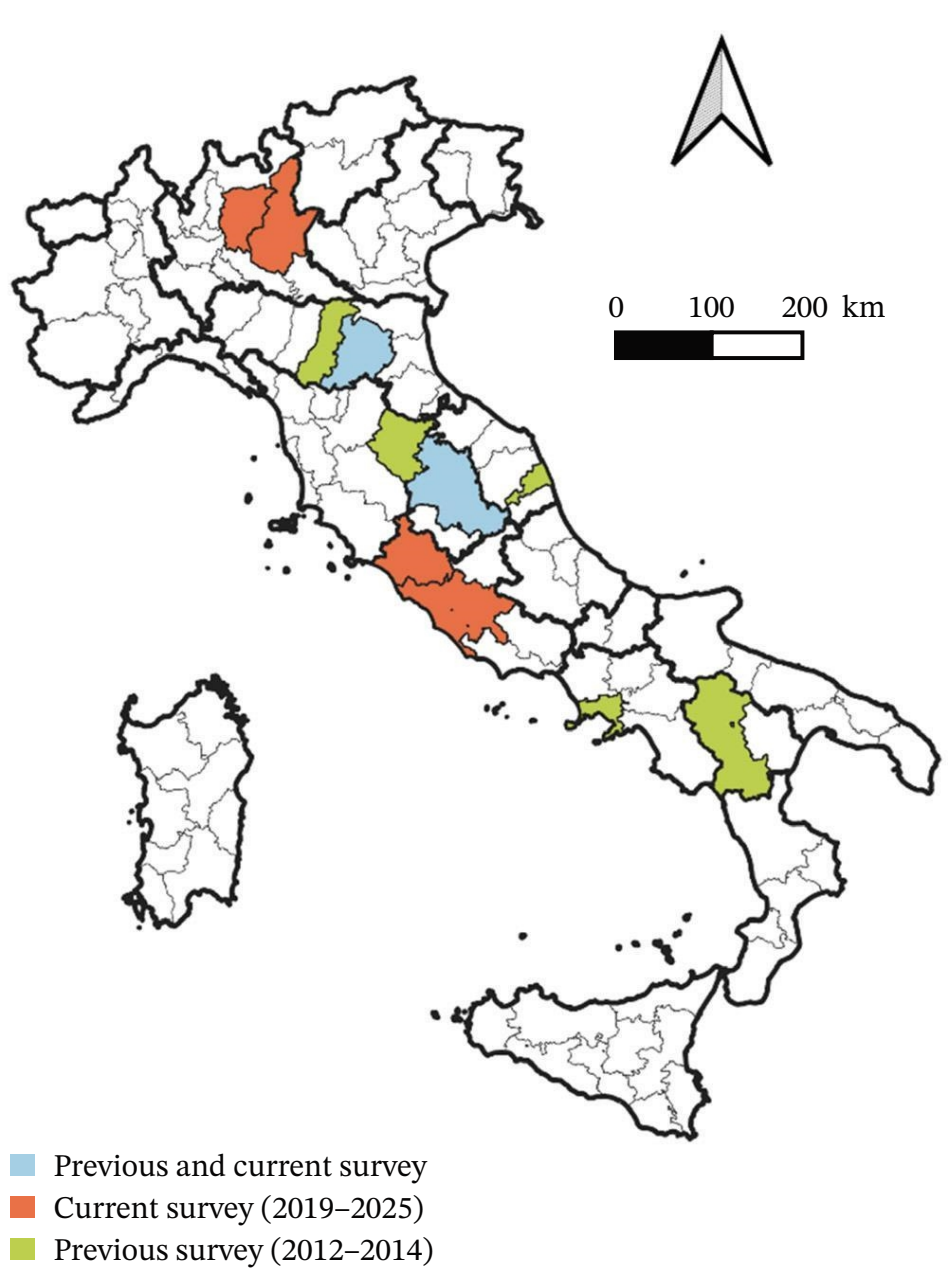
Location of the provinces where the farms were sampled.

Each pooled sample was prepared by combining 10 spatially distinct fresh fecal deposits collected from the floor of the same pen or, when necessary, in pens with less than 20 pigs, in neighboring pens. Fecal deposits were collected ideally immediately after defecation, with at least 10 g of fecal material obtained from each deposit. Because animals were not individually observed during defecation, it cannot be excluded that some feces originated from the same animal. Moreover, we cannot exclude that the same pig contributed fecal material to more than one pooled sample. The combined samples were then placed into a sterile plastic bag using disposable plastic spoons. Gloves and sampling tools were replaced between the collections of each pooled sample to prevent contamination. The pooled samples were stored at −20°C until further analysis. A total of 20 pooled samples were analyzed per herd. Across the 12 investigated farms, 10 were fattening farms, with sampled pigs distributed across the 30–100, 101–140, and >140‐day age classes; one (IT60) was a weaning unit with sampled pigs aged 30–100 days, and one (IT12) was a farrow‐to‐finish farm where fecal samples were collected from animals between 30 and 100 and >140‐day age classes (Table 1). Nine farms out of the 12 investigated were affiliated with a production supply chain.

**Table 1:** Detection of the investigated viruses by farm, the corresponding farm type and age class are reported.

Farm	Type of farms	Age class	No. of positive pooled samples						
			HEV	RVA	KoV	AstV	CoV	CaliciV^a^	Total
IT10	Fattening	>140	0	6	9	11	0	0	26
IT11	Fattening	>140	0	2	15	17	0	0	34
IT12	F to F^b^	30–100 and >140	13	4	0	17	0	0	34
IT15	Fattening	101–140 and >140	14	7	16	16	0	3	56
IT17	Fattening	>140	0	1	13	0	0	0	14
IT20	Fattening	101–140	9	13	17	19	0	0	58
IT52	Fattening	101–140 and >140	5	5	1	10	0	0	21
IT56	Fattening	101–140	16	0	0	20	0	0	36
IT57	Fattening	30–100	8	0	3	0	0	0	11
IT58	Fattening	101–140	0	0	20	18	0	0	38
IT59	Fattening	30–100	0	1	15	20	0	0	36
IT60	Weaning	30–100	0	8	14	20	0	0	42
Total pooled samples positive (%)^c^			65 (27.1)	47 (19.6)	123 (51.2)	168 (70.0)	0	3 (1.3)	406
Positive farms (%)			6 (50.0)	9 (75.0)	10 (83.3)	10 (83.3)	0	1 (8.3)	—

^a^Calicivirus.

^b^Farrow to finish.

^c^Percentage of positive pooled samples out of total samples (*N* = 240).

The pooled swine fecal samples were tested for the presence of AstV, NoV, SaV, KoV, RVA, CoV, and HEV RNA.

### 2.2. RNA Extraction

One hundred milligrams of homogenized fecal material were mixed thoroughly by vortexing in RNase‐free water to a final concentration of 10% (w/v). Following centrifugation at 10,000 × g for 10 min, the clear fecal supernatant was collected. From this, 140 μL was used for RNA extraction using the QiaCube automated extraction system in combination with the QIAamp Viral Mini kit (Qiagen, Milan, Italy), following the manufacturer’s protocol, except for the final elution volume, which was set to 100 μL. The extracted RNA was stored at −80°C until use.

### 2.3. Viral Nucleic Acid Detection

Real‐time reverse transcription (RT)‐PCR was used for the detection of HEV and RVA. Samples were tested using the QuantiFast Pathogen PCR + IC Kit (Qiagen, Hilden, Germany), following the procedures described previously (Table 2).

**Table 2:** Methodology description and list of the primers and probes sequences employed for the detection and sequencing of the investigated viruses.

Virus family	Target virus	Assay	Primers/probes (5′–3′ sequence)	References
Hepeviridae	HEV	RT‐qPCR	JVHEVF: GGTGGTTTCTGGGGTGAC JVHEVR: AGGGGTTGGTTGGATGAA JVHEVP‐MGB: FAM‐TGATTCTCAGCCCTTCGC‐MGB	[44]
		RT‐PCR	HE044: CAA GGH TGG CGY TCK GTT GAG AC HE040: CCC TTR TCC TGC TGA GCR TTC TC	[45]
		Nested‐PCR	HE110‐2^a^: GYT CKG TTG AGA CCT CYG GGG T GYT CKG TTG AGA CCA CGG GYG T YT CKG TTG AGA CCT CTG GTG T HE041: TTM ACW GTC RGC TCG CCA TTG GC	[45]

Coronaviridae	CoV	RT‐PCR	Pan_CoV_F‐1: GGTGGGAYTAYCCHAARTGYGA Pan_CoV_R‐1: CCRTCATCAGAHARWATCAT Pan_CoV_R‐2: CCRTCATCACTHARWATCAT	[46]
		Semi‐nested PCR	Pan_CoV_F‐2: GAYTAYCCHAARTGTGAYAGA Pan_CoV_F‐3: GAYTAYCCHAARTGTGAYMGH Pan_CoV_R‐1: CCRTCATCAGAHARWATCAT Pan_CoV_R‐2: CCRTCATCACTHARWATCAT	[46]

Caliciviridae	SaV/NoV	RT‐PCR	p289: TGA CAA TGT AAT CAT CAC CAT A p290: GAT TAC TCC AAG TGG GAC TCC AC	[47]

Reoviridae	RVA	RT‐qPCR	JVKF: CAGTGGTTGATGCTCAAGATGGA JVKR: TCATTGTAATCATATTGAATACCCA JVKP: FAM‐ACAACTGCAGCTTCAAAAGAAGWGT‐BHQ	[48]
		RT‐PCR	VP7‐F: ATG TAT GGT ATT GAA TAT ACC AC VP7‐R: AAC TTG CCA CCA TTT TTT CC VP4‐F: TAT GCT CCA GTN AAT TGG VP4‐R: ATT GCA TTT CTT TCC ATA ATG	[49]

Picornaviridae	KoV	RT‐PCR	UNIV‐kobu‐F: TGGAYTACAAG(/R)TGTTTTGATGC UNIV‐kobu‐R: ATGTTGTTRATGATGGTGTTGA	[50]

Astroviridae	AstV	RT‐PCR	PAstV‐F: TGACATTTTGTGGATTTACAGTT PAstV‐R: CACCCAGGGCTGACCA	[51]

*Note:* Degenerate nucleotide positions: H = A, T, or C; Y = T or C; K = G or T; M = A or C; W = A or T.

^a^The sense primer HE110‐2 consists of a mixture of three sequences.

For the detection of KoVs, AstVs, CoVs, NoVs, and SaVs, RT followed by conventional end‐point PCR was performed. RT was carried out using SuperScript III Reverse Transcriptase (Life Technologies, Carlsbad, CA, USA) with randomly tagged primers, and the first round of PCR was performed using the GoTaq G2 DNA polymerase (Promega, Madison, WI, USA) with specific primer sets. When applicable, a second round of nested PCR was also conducted, following protocols described earlier (Table 2).

Additionally, RT followed by end‐point PCR was used to generate amplicons from RNA samples that tested positive by real‐time PCR performed for HEV and RVA detection (Table 2).

### 2.4. DNA Sequencing

Due to financial constraints, only 70 nucleotide sequencing analyses were performed in the present study. Up to three positive samples per farm, if detected, were randomly selected for each viral pathogen and sequenced. The expected‐size PCR amplicons were confirmed via Sanger sequencing performed using a custom service (Eurofins Genomics, Ebersberg, Germany). The resulting nucleotide sequences were edited and aligned using MEGA software. Nucleotide identity searches were conducted using the BLASTn tool on the NCBI GenBank database (http://www.ncbi.nlm.nih.gov/genbank/). The obtained nucleotide sequences were submitted to NCBI and are available under the following Accession Numbers: PX636894 (SaV); PX653487‐PX653514 (AstV); PX653515‐PX653516 (NoV); PZ027504‐PZ027513 (RVA); PZ027514‐PZ027538 (KoV); and PX626018‐26 (HEV).

Genotypes and subtypes of NoV and HEV were determined using the online NoV Typing Tool (http://www.rivm.nl/mpf/norovirus/typingtool) and HEVnet typing tool (https://www.rivm.nl/mpf/typingtool/hev/), respectively. The maximum likelihood (ML) phylogenetic trees were constructed as suggested by the MEGA6 software model test (http://www.megasoftware.net) based on 1000 bootstrap replications.

### 2.5. Statistical Analysis

The observational unit of the study was the pooled fecal sample. Accordingly, the results were expressed as pool‐level detection frequencies.

Associations between the detection of each virus and animal‐ and farm‐level characteristics were assessed using logistic regression models based on generalized estimating equations (GEEs) to account for the correlation among pooled samples originating from the same farm. GEE models were not fitted for CoV because no positive pools were detected, nor for caliciviruses because only three positive pools were identified, precluding reliable estimation. Farm was specified as the clustering variable, and an exchangeable within‐cluster correlation structure was assumed.

For each virus, separate GEE models were fitted considering the following explanatory variables: pig age class (30–100, 101–140, and >140 days), farm affiliation with a production supply chain, and herd size (≤1000 or >1000 pigs).

The reference categories were 30–100 days for age, no affiliation with a production supply chain, and herd size ≤1000 pigs. Given the limited number of independent clusters (12 farms), bias‐reduced robust covariance estimates for small samples were used for statistical inference. Results are reported as odds ratios (ORs), 95% CIs, and *p*‐values. For age, which comprised three categories, the overall effect was assessed using a 2‐degree‐of‐freedom Wald test. Statistical significance was set at *p*  < 0.05.

As a secondary analysis, viral codetection was evaluated and defined as the detection of RNA from at least two of the investigated viruses within the same pooled sample. This variable was analyzed as a binary outcome (0–1 virus vs. ≥2 viruses) using the same GEE approach.

Statistical analyses were performed using IBM SPSS Statistics version 30.0 (IBM SPSS Statistics, New York, NY, USA). GEE analyses with bias‐reduced robust covariance estimation were conducted through Python integration.

## 3. Results

### 3.1. Detection and Distribution of Investigated Viruses

Considering at least one pooled fecal sample positive among the analyzed viruses, all viruses except CoV were detected in at least one farm. AstV and KoV were the most widespread viruses, each identified in 10 of 12 farms (83.3%), followed by RVA in 9 farms (75.0%) and HEV in 6 farms (50.0%). Caliciviruses were detected in only one farm (8.3%) (Table 1). In some farms, multiple viruses were detected simultaneously, with several cases showing a high proportion of positive pools among the 20 examined. In farms IT20 and IT15, four viruses were identified in 7 and 4 pools, respectively, resulting in 58 and 56 positive detections (Table 1). Conversely, IT17 and IT57 exhibited the lowest total numbers of viral detections, 14 and 11, respectively (Table 1).

The most frequently detected viruses were KoV and AstV, present each in 10 out of the 12 farms. KoV RNA was found in 123/240 pooled fecal samples (51.2%); within farms, the number of positive samples varied between 1/20 and 20/20. PAstV was detected in 168/240 pooled fecal samples (70.0%) with farm‐level detection ranging from 10/20 to 20/20 pools. HEV RNA was detected in 27.1% of pooled samples (65/240) across six farms, three of which were previously investigated [35]. The HEV positivity ranged from 5/20 to 16/20 pools. RVA RNA was detected in 47/240 samples (19.6%) and was found in nine farms, with between 1/20 and 13/20 pools testing positive. Calicivirus RNA was detected in only one farm (3/240; 1.3%). No pooled sample tested positive for CoV (Table 1).

To provide a descriptive comparison with the previous survey, the main characteristics and viral detection results of the two studies are summarized in Table 3.

**Table 3:** Main characteristics and viral detection results of the previous and current surveys.

Characteristic	Previous survey (2012–2014)^a^	Current survey (2019–2025)
Number of farms	8	12
Geographical areas	Northern, Central, and Southern Italy	Northern and Central Italy
Number of samples	242	240
Type of samples	Individual fecal samples	Pooled fecal samples
AstV	163/242 (67.4%)	168/240 (70.0%)
HEV	45/242 (18.6%)	65/240 (27.1%)
RVA	10/242 (4.1%)	47/240 (19.6%)
NoV/SaV	0/242 (0%)	3/240 (1.3%)^b^
PKoV	Not investigated	123/240 (51.2%)
CoV	Not investigated	0/240 (0%)

^a^Percentages in the previous survey refer to individual‐animal prevalence, whereas percentages in the current survey represent pool‐level detection frequencies.

^b^Sequencing identified one SaV and two NoV strains.

Out of the 240 samples tested, 207 (86.3%) were positive for at least one virus. Among these, 59 samples (24.6%) contained RNA from a single virus; AstV was detected in 27 pools (11.3%), HEV in 9 (3.8%), and KoV in 23 (9.6%). Overall, 148 pools (61.7%) contained RNA from at least two viruses (Table 4). In 108 samples (45.0%), exactly two viruses were concurrently detected within the same pooled sample. Three viruses were detected concurrently in 29 samples (12.1%) and four viruses in 11 samples (4.6%) (Table 4).

**Table 4:** Distribution of pooled samples by number of viruses detected per pool.

No. of viruses detected	No. of pooled samples (%)
0	33 (13.8)
1	59 (24.6)
2	108 (45.0)
3	29 (12.1)
4	11 (4.6)
Total	240 (100)

As described in Table 5, the most frequent viral codetection pattern was AstV and KoV, identified in 62 pools (25.8%) and in 7 farms (58.3%), followed by HEV and AstV (29 samples, 12.1%; 41.7% of farms) and RVA + AstV (4.6% of pools; 58.3% of farms). Triple and quadruple combinations were less frequently observed (Table 5).

**Table 5:** Detection rates of the multiple virus combinations (2–4) observed in pooled samples.

Virus combination	No. of positive pools (%)	No. of positive farms (%)
HEV + AstV	29 (12.1)	5 (41.7)
HEV + AstV + CaliciV	1 (0.4)	1 (8.3)
HEV + KoV	1 (0.4)	1 (8.3)
HEV + KoV + AstV	6 (2.5)	2 (16.7)
HEV + KoV + AstV + CaliciV	1 (0.4)	1 (8.3)
HEV + RVA	1 (0.4)	1 (8.3)
HEV + RVA + AstV	6 (2.5)	2 (16.7)
HEV + RVA + KoV	1 (0.4)	1 (8.3)
HEV + RVA + KoV + AstV	10 (4.2)	2 (16.7)
KoV + AstV	62 (25.8)	7 (58.3)
KoV + AstV + CaliciV	1 (0.4)	1 (8.3)
RVA + AstV	11 (4.6)	7 (58.3)
RVA + KoV	4 (1.7)	2 (16.7)
RVA + KoV + AstV	14 (5.8)	4 (33.3)

### 3.2. Factors Associated With Viral Detection and Codetection

The pool‐level detection frequencies and results of the farm‐adjusted GEE models are reported in Table 6.

**Table 6:** Pool‐level viral RNA detection according to age class and farm characteristics: farm‐adjusted GEE analysis.

Virus	Variable	Category	Positive pools/total (%)	OR	95% CI	*p*‐Value	Overall *p*
HEV	Age class	30–100 days	18/74 (24.3)	1.00	Ref.	—	0.701
		101–140 days	34/71 (47.9)	3.36	0.14–83.06	0.459	—
		>140 days	13/95 (13.7)	0.46	0.06–3.63	0.459	—
	Supply chain	No	32/60 (53.3)	1.00	Ref.	—	—
		Yes	33/180 (18.3)	0.20	0.03–1.37	0.100	—
	Herd size	≤1000 pigs	27/120 (22.5)	1.00	Ref.	—	—
		>1000 pigs	38/120 (31.7)	1.60	0.21–12.39	0.655	—

RVA	Age class	30–100 days	12/74 (16.2)	1.00	Ref.	—	0.587
		101–140 days	19/71 (26.8)	2.46	0.30–19.79	0.399	—
		>140 days	16/95 (16.8)	0.78	0.17–3.67	0.754	—
	Supply chain	No	16/60 (26.7)	1.00	Ref.	—	—
		Yes	31/180 (17.2)	0.57	0.17–1.98	0.377	—
	Herd size	≤1000 pigs	31/120 (25.8)	1.00	Ref.	—	—
		>1000 pigs	16/120 (13.3)	0.44	0.08–2.50	0.355	—

PKoV	Age class	30–100 days	32/74 (43.2)	1.00	Ref.	—	0.602
		101–140 days	40/71 (56.3)	0.84	0.27–2.57	0.755	—
		>140 days	51/95 (53.7)	1.29	0.63–2.64	0.482	—
	Supply chain	No	17/60 (28.3)	1.00	Ref.	—	—
		Yes	106/180 (58.9)	3.62	0.15–88.90	0.430	—
	Herd size	≤1000 pigs	55/120 (45.8)	1.00	Ref.	—	—
		>1000 pigs	68/120 (56.7)	1.55	0.23–10.40	0.654	—

PAstV	Age class	30–100 days	53/74 (71.6)	1.00	Ref.	—	0.762
		101–140 days	64/71 (90.1)	0.44	0.002–83.84	0.759	—
		>140 days	51/95 (53.7)	0.33	0.02–6.28	0.462	—
	Supply chain	No	43/60 (71.7)	1.00	Ref.	—	—
		Yes	125/180 (69.4)	0.90	0.14–5.88	0.911	—
	Herd size	≤1000 pigs	74/120 (61.7)	1.00	Ref.	—	—
		>1000 pigs	94/120 (78.3)	2.25	0.20–24.97	0.510	—

Detection frequencies represent the proportion of pooled fecal samples testing positive.

No statistically significant associations were identified between the detection of the investigated viruses and the animal‐ or farm‐level characteristics considered (Table 6). Detection frequencies varied across age classes, with the highest values generally observed in pigs aged 101–140 days for HEV (47.9%), RVA (26.8%), and PAstV (90.1%), while PKoV (56.3%) detection remained relatively high across all age groups; however, age was not significantly associated with detection of any virus (overall *p*‐values 0.587–0.762).

Differences according to production supply chain affiliation were observed for HEV and PKoV, with higher HEV detection in nonaffiliated farms (53.3% vs. 18.3%) and higher PKoV detection in affiliated farms (58.9% vs. 28.3%), whereas RVA and PAstV frequencies were more similar between affiliated and nonaffiliated farms (RVA: 17.2% vs. 26.7%; PAstV: 69.4% vs. 71.7%, respectively). Nevertheless, none of the associations reached statistical significance (*p* = 0.100–0.911). Likewise, no significant association was observed with herd size. The wide confidence intervals for several estimates indicate substantial uncertainty around these estimates.

The frequency of codetection of at least two viruses was 56.8% in the 30–100‐day age class, 87.3% in the 101–140‐day class, and 46.3% in pigs aged >140 days. Although codetection was descriptively highest in the 101–140‐day age class, the overall association with age was not statistically significant in the GEE model (*p* = 0.544).

Codetection was observed in 65.0% of pools from non‐supply‐chain farms and 60.6% of those from supply‐chain farms (OR = 0.83, 95% CI: 0.09–7.82; *p* = 0.868). A higher codetection frequency was observed in farms with >1000 pigs than in those with ≤1000 pigs (70.8% vs. 52.5%), but the association was not statistically significant (OR = 2.20, 95% CI: 0.34–14.21; *p* = 0.408) (Table 7).

**Table 7:** Codetection of at least two viruses within the same pooled fecal sample: farm‐adjusted GEE analysis.

Variable	Category	≥2 viruses/total (%)	OR	95% CI	*p*‐Value	Overall *p*
Age class	30–100 days	42/74 (56.8)	1.00	Ref.	—	0.544
	101–140 days	62/71 (87.3)	4.20	0.33–53.63	0.270	—
	>140 days	44/95 (46.3)	0.78	0.17–3.52	0.745	—
Supply chain	No	39/60 (65.0)	1.00	Ref.	—	—
	Yes	109/180 (60.6)	0.83	0.09–7.82	0.868	—
Herd size	≤1000 pigs	63/120 (52.5)	1.00	Ref.	—	—
	>1000 pigs	85/120 (70.8)	2.20	0.34–14.21	0.408	—

### 3.3. Nucleotide Sequence Analysis

The sequence analyses of a 700‐bp fragment (covering the ORF1b‐ORF2 region) from 28 AstV strains collected across 10 farms were conducted. Phylogenetic analyses were performed separately on the partial nucleotide sequences of the AstV capsid protein gene and on the partial RNA‐dependent RNA polymerase (RdRp) gene (Supporting Information 1: Figure S1 and Supporting Information 2: Figure S2). All but one of the analyzed strains were classified within the PAstV group 4, exhibiting wide nucleotide sequence heterogeneity with a nt.id of up to 95%. One strain was classified as PAstV2. Any clear association between genetic grouping and the specific farm of origin was observed.

Hepatitis E virus (HEV) was typed using a 300 bp fragment within the ORF2 region, which encodes the capsid protein. All sequenced strains belonged to genotype HEV‐3, with 7 sequences obtained from 7 farms previously published [52] together with 9 sequences obtained in the present study, showing the subtype 3f as predominant with the sporadic detection of subtypes 3e and 3c, detected in one farm each. The 3f sequences were further divided into two subclusters, designated 3f1 and 3f2 (Supporting Information 3: Figure S3). Among the samples sequenced within each farm, only one sequenced strain (100% nt id.) was retrieved from each farm. The analyses also confirmed correlation with other strains belonging to the HEV‐3f subtype already detected in Italy in both humans, pigs, and wild boar.

Only one SaV strain was sequenced from farm IT15. Sequence typing based on the RdRp fragment confirmed its classification as genotype VIII. The retrieved sequence showed low similarity to other sequences available online, displaying up to 85% nt id. within the same Caliciviridae family. From the same farm that tested positive for SaV (Supporting Information 4: Figure S4), two sequences matching NoV genotype GII.11 were also retrieved. The two strains from one farm were related to a group of sequences (MN605609; MN605610; MN605625) detected in pigs, in a farm located in Northern Italy in 2018 (Supporting Information 5: Figure S5).

Twenty‐five KoV‐positive samples were subjected to nucleotide sequencing of the 3D polymerase protein gene. The obtained nucleotide sequences were all found to belong to the PKoV genotype, but they showed a high degree of heterogeneity among themselves, with nucleotide identities ranging between 92.5% and 100% (Supporting Information 6: Figure S6). In addition, the geographical distribution of the KoV strains previously detected and related to the sequences obtained in this study was heterogeneous, since they were isolated worldwide.

RVA‐positive samples were subjected to nucleotide sequencing, which allowed the obtain 10 sequences of the VP7 protein gene. The VP7 genotypes detected were G2 (*n* = 4), G5 (*n* = 4), G9 (*n* = 1), and G11 (*n* = 1). Three G2 strains were related to human RVA belonging to the same genotype, while strains belonging to G5, G9, and G11 genotypes were related to swine RVA strains. The sequences obtained were detected in 6 farms. Farms IT‐10, IT‐11, IT‐15, and IT‐52 revealed the circulation of a single genotype within the farm (G2, G5, G2, and G9, respectively), while farms IT‐12 and IT‐20 showed the cocirculation of 2 genotypes (G5 + G11 and G2 + G5, respectively) (Supporting Information 7: Figure S7).

## 4. Discussion

Major outbreaks of viral diseases remain a persistent and significant challenge in the pig industry, with gastrointestinal diseases being among the most common in pigs, involving especially the youngest. Moreover, approximately half of the pathogens detected in swine are viruses with zoonotic potential and can be transmitted to humans through direct or indirect contacts [53].

Ten years ago, we conducted a survey to evaluate the circulation of several viruses, such as RVA, HEV, AstV, and Caliciviruses, within swine farms located in different Italian geographical areas [43]. The previous survey documented widespread detection of these viruses in clinically healthy pigs. To assess the circulation of these groups of enteric viruses in pig farms from Northern and Central Italy, areas also investigated approximately 10 years earlier, and at the same production stage (fatteners), and to descriptively compare the current findings with those of the previous survey [43], we conducted a follow‐up study. The viral panel was also expanded to include PKoV and CoVs.

As it was not possible to investigate the same farms, the comparison was intended to identify broad patterns of persistence and circulation in the same geographical areas rather than to quantify temporal trends. The study also aimed to evaluate whether viral detection was associated with pig age and selected farm characteristics while accounting for the clustered sampling design. Results of the present study confirmed the high detection frequencies of investigated enteric viruses in the 12 Italian swine farms investigated, with possible important implications for both animal health and zoonotic risk.

Hepatitis E virus (HEV), an established zoonotic pathogen, was detected in 65/240 (27.1%) of pooled fecal samples, including only strains of genotype 3f. Subtype 3f is one of the prevalent subtypes causing infection in humans in Italy [50]. In our previous study, HEV was detected in 18.6% of individual samples. The higher pool‐level detection frequency observed in the present study can be due to many factors, including the diagnostic methods employed in this study. In fact, the real‐time RT‐PCR assay, instead of the nested RT‐PCR used previously, can be more sensitive. Differences in the sampled farms or temporal epidemiological variation cannot be excluded. HEV detection was descriptively the highest in the 101–140‐day age class (47.9%) and in non‐supply chain farms (53.3% vs. 18.3%), although neither association was statistically significant.

PAstV was the most frequently detected virus also in our previous survey [43], and the actual detection rate (70% of pooled samples and 83% of farms) is comparable to results reported previously [3], confirming its widespread circulation in pig herds and supporting the hypothesis of its endemic presence. Detection was descriptively the highest in pools from 101–140‐day‐old pigs (90.1%) and in farms with the highest population (78.3% vs. 61.7%). These differences should therefore be interpreted as descriptive patterns rather than evidence of an effect of herd size. PKoV was detected in 51.2% of fecal pools across 10 of the 12 farms. This finding is in line with our previous report in Italy, which identified positive samples in six farms, with prevalence ranging from 24% to 84% [54]. It is also consistent with reports from other European countries, where detection rates range between 30% and 85%, depending on age and sampling methods [2, 4, 5, 27]. The detection of the virus in asymptomatic animals, its ability to infect a wide range of species, and its capacity to undergo recombination events raise concern about the emergence of more severe symptoms in pigs, supporting the need for continued virological surveillance [55].

RVA was detected in 47/240 (19.6%) of pooled samples from 9 out of 12 farms; this pool‐level detection frequency was higher than the 4.1% individual‐level prevalence reported in the previous survey; however, the two results are not directly comparable due to differences in the sampling strategy. RVA is common among farms (9/12), however, fewer than seven pooled samples (except for two farms) tested positive within each farm. The genotypes detected included the G2 and G9 genotypes, two major RVA genotypes able to infect both pigs and humans, and interestingly, three G2 sequences obtained in this study were strictly related to human G2 RVA strains. Moreover, RVA circulation in pigs is relevant to swine health, as RVA is a major cause of diarrhea in young animals, and its capacity for genetic reassortment and its genetic relatedness to human strains also support the need for sequencing to monitor genetic variation among circulating strains [31].

NoV and SaV RNA were detected in only one farm, in 3 pooled fecal samples (1.3%), with sequencing identifying two NoV strains and one SaV strain. This low detection frequency is consistent with the earlier survey [43] and with previous studies reporting a sporadic occurrence of porcine calicivirus in pig herds [3, 23, 27]. The detection of only one SaV‐positive pool suggests a low occurrence of SaV among asymptomatic pigs, even if, in our previous study, a 6.9% prevalence was reported [22]. In Italy, an extensive study reported 11.4% of fecal samples positive for porcine NoV, originating from 22 farms [19], indeed confirming the limited circulation of the virus in pigs.

CoVs, included for the first time in this survey, were not detected in any of the pooled fecal samples. The absence of CoV detection in the present study may reflect a limited circulation in the sampled herds during the study period. Nevertheless, this finding should be interpreted with caution since only 12 farms were investigated, not being representative of the whole country. Results are consistent with previous European findings, showing low seroprevalence of PEDV, TGEV, and especially PRCV and rare molecular detection in both domestic pigs and wild boar [5, 56, 57]. Recent Italian surveys report modest antibody prevalence (14.8% PEDV, 5.5% TGEV, and 0.9% PRCV) [56], while PEDV prevalence remains very low in Europe [5, 57]. Sampling, involving mainly older pigs, which shed less virus, and analysis of fecal material for targeting nonenteric CoVs may also have contributed to the absence of detection.

AstV, RVA, and HEV sequences confirmed the circulation of different strains. AstV (for which more sequences were retrieved), differently from the other viruses, showed the simultaneous circulation of different viral strains within the same farms. This is not surprising because, in addition to the use of pooled samples, which can hinder the detection of less represented strains, different AstV types are widely reported to coinfect the same animal. As previously described, only one strain of HEV circulates within each farm [43]. Interestingly, SaV and NoV sequences showed limited similarity with other sequences on the NCBI database, suggesting high heterogeneity of circulating strains or limited reference data, but more interestingly, this finding could highlight the detection of novel viral variants. Both NoV sequences matched with GII.11, a typical swine genotype. While three genotypes have been described in pigs, in Italy, GII.11 is the most frequent detected, followed by GII.18. The first identification of GII.11 in Italy occurred in 2014 [22], and only more recently, GII.18 was identified [19]. Although the epidemiological significance of the different swine NoVs remains poorly defined, genotype GII.11 is reported as one of the most detected in pigs [18]. A study limitation was the use of the highly conserved RdRp region for calicivirus and KoV, which are not fully informative for strain characterization, but it was chosen because it is highly sensitive.

Concurrent detection of multiple viruses within the same pooled fecal sample was common: 148/240 pools (61.7%) contained RNA from two or more viruses, and among the remaining pools, the most frequent viral combination detected was PAstV and PKoV. This is partially linked to the use of pooled samples, as the detection of multiple viruses within the same pool may reflect infections in different animals. However, coinfection of a single animal cannot be excluded, and some viral combinations could cause more severe symptoms in affected animals [5, 58]. Recent metagenomic studies revealed that the porcine gut hosts a complex and diverse virome, including eukaryotic viruses and bacteriophages [59], which may significantly influence host health, particularly in young pigs [60]. Our findings align with previous studies confirming that pigs harbor a wide range of viral species, many of which may persist as subclinical or endemic infections [61, 62]. Understanding this viral diversity is important for managing pig health and assessing the potential risk associated with the emergence of novel strains.

The present surveillance provides useful information on the circulation of enteric viruses in the investigated pig population. However, understanding whether viral detection patterns have changed over time would also require information on changes in farm management and biosecurity practices. Over the last decade, increasing attention has been given to biosecurity in European pig production, including measures aimed at preventing the introduction and within‐farm spread of infectious agents [63]. Such changes may have contributed to changes in the circulation of enteric viruses. However, this could not be assessed in the present study because the same farms investigated in previous surveys were not included, and changes in biosecurity and management practices over time were not recorded in the 12 investigated farms. Therefore, differences between surveys should be interpreted cautiously, but factors such as changes in the biosecurity measures, also for the recent African swine fever crisis, cannot be excluded.

This study provides important insights, although it has some limitations that should be considered when interpreting the findings and the differences between the two surveys. Only 12 farms were investigated, and the study relies on limited geographic coverage; the use of pooled fecal samples allows assessment of virus circulation at the farm level rather than individual prevalence. Studying the association of virus frequency with age and farm management was limited, indeed not significant, to allow comparison with our previous survey [43]. Although the conventional RT‐PCR assays used in this study are established methods for molecular screening, real‐time RT‐PCR offers important advantages for surveillance, particularly with respect to quantitative assessment of virus present, in particular in the coinfection.

However, the results demonstrate that enteric viruses are widely distributed among the sampled farms, with AstV and KoV being the most frequently detected viruses, while HEV and RVA were detected in several farms, caliciviruses were uncommon, and CoV RNA was not detected under the specific sampling and assay conditions used; in addition, only fecal samples were tested, whereas porcine CoVs are primarily associated with respiratory disease.

Among the viruses detected, HEV has an established zoonotic role, whereas the zoonotic significance of PAstV, PKoV, porcine NoV, SaV, and RVA strains identified remains unclear. Future control strategies should combine strengthened farm biosecurity with longitudinal molecular surveillance. Measures aimed at limiting fecal–oral transmission may contribute to reducing infection pressure. Longitudinal farm‐based sampling, sensitive molecular assays, phylogenetic characterization, and systematic collection of biosecurity and management data would also allow for more reliable evaluation of temporal changes in viral circulation and of the effectiveness of specific farm‐level interventions.

## Funding

This work was cofunded by the European Union’s Horizon Europe Project (Grant 101136346) EUPAHW.

## Disclosure

Views and opinions expressed are, however, those of the authors only and do not necessarily reflect those of the European Union or the European Research Executive Agency. Neither the European Union nor the granting authority can be held responsible for them.

## Ethics Statement

Ethical approval was not required for this study as it did not involve experimental procedures, animal handling, or invasive interventions. Fecal samples were collected from the ground shortly after spontaneous defecation. No additional procedures were performed, and animal welfare was not compromised.

## Conflicts of Interest

The authors declare no conflicts of interest.

## Supporting Information

Additional supporting information can be found online in the Supporting Information section.

## Supporting information


**Supporting Information 1** Figure S1: Phylogenetic tree based on the astrovirus (AstV) capsid protein gene partial ORF nucleotide sequences. The AstV strains sequenced in this study are highlighted with a filled circle. The maximum likelihood (ML) phylogenetic tree was constructed with the Kimura‐2 with gamma distributed and uniform rates parameter model (K2+G), with 1000 bootstrap repetitions. Bootstrap values under 70% are not shown.

**Supporting Information 2** Figure S2: Phylogenetic tree based on the astrovirus (AstV) RdRp protein gene partial ORF nucleotide sequences. The AstV strains sequenced in this study are highlighted with a filled circle. The maximum likelihood (ML) phylogenetic tree was constructed with the Kimura‐2 with gamma distributed and uniform rates parameter model (K2+G), with 1000 bootstrap repetitions. Bootstrap values under 70% are not shown.

**Supporting Information 3** Figure S3: Phylogenetic tree based on the hepatitis E virus (HEV) partial ORF2 gene nucleotide sequences. The HEV strains sequenced in this study are highlighted with a filled circle. HEV strains previously sequenced are highlighted with an empty circle. The maximum likelihood (ML) phylogenetic tree was constructed with the Tamura‐Nei with gamma distributed and invariant sites parameter model (TN93+G+I), with 1000 bootstrap repetitions. Bootstrap values under 70% are not shown.

**Supporting Information 4** Figure S4: Phylogenetic tree based on the sapovirus (SaV) RdRp protein gene partial ORF nucleotide sequences. The SaV strains sequenced in this study are highlighted with a filled circle. The maximum likelihood (ML) phylogenetic tree was constructed with the Kimura‐2 with gamma distributed and uniform rates parameter model (K2+G), with 1000 bootstrap repetitions. Bootstrap values under 70% are not shown.

**Supporting Information 5** Figure S5: Phylogenetic tree based on the norovirus (NoV) RdRp protein gene partial ORF nucleotide sequences. The NoV strains sequenced in this study are highlighted with a filled circle. The maximum likelihood (ML) phylogenetic tree was constructed with the Kimura‐2 with gamma distributed and uniform rates parameter model (K2+G), with 1000 bootstrap repetitions. Bootstrap values under 70% are not shown.

**Supporting Information 6** Figure S6: Phylogenetic tree based on the swine kobuvirus (KoV) partial ORF of the 3D polymerase protein gene nucleotide sequences. The KoV strains sequenced in this study are highlighted with a filled circle. The maximum likelihood (ML) phylogenetic tree was constructed with the Kimura‐2 with gamma distributed and uniform rates parameter model (K2+G), with 1000 bootstrap repetitions. Bootstrap values under 70% are not shown.

**Supporting Information 7** Figure S7: Phylogenetic tree based on the partial ORF of the VP7 protein gene (gene 9) of group A rotavirus (RVA) nucleotide sequences. The RVA strains sequenced in this study are highlighted with a filled circle. The maximum likelihood (ML) phylogenetic tree was constructed with the Tamura‐3 with gamma distributed and invariant sites parameter model (T92+G+I), with 1000 bootstrap repetitions. Bootstrap values under 70% are not shown.

## Data Availability

The nucleotide sequences of the viral strains identified in this study are available in GenBank (https://www.ncbi.nlm.nih.gov/genbank/) under the following Accession Numbers: PX636894 (SaV); PX653487‐PX653514 (AstV); PX653515‐PX653516 (NoV); PZ027504‐PZ027513 (RVA); PZ027514‐PZ027538 (KoV); and PX626018‐26 (HEV).
