# Supplementary material for: Ten Years Later: Molecular Detection and Circulation of Enteric Viruses in Italian Pig Herds
Source: Transbound Emerg Dis. 2026 Oct 30;2026:4011873. doi: 10.1155/tbed/4011873 (PMC13721171; doi:10.1155/tbed/4011873)
Supplement: Supplementary file 1 [file TBED-2026-4011873-s001.docx]

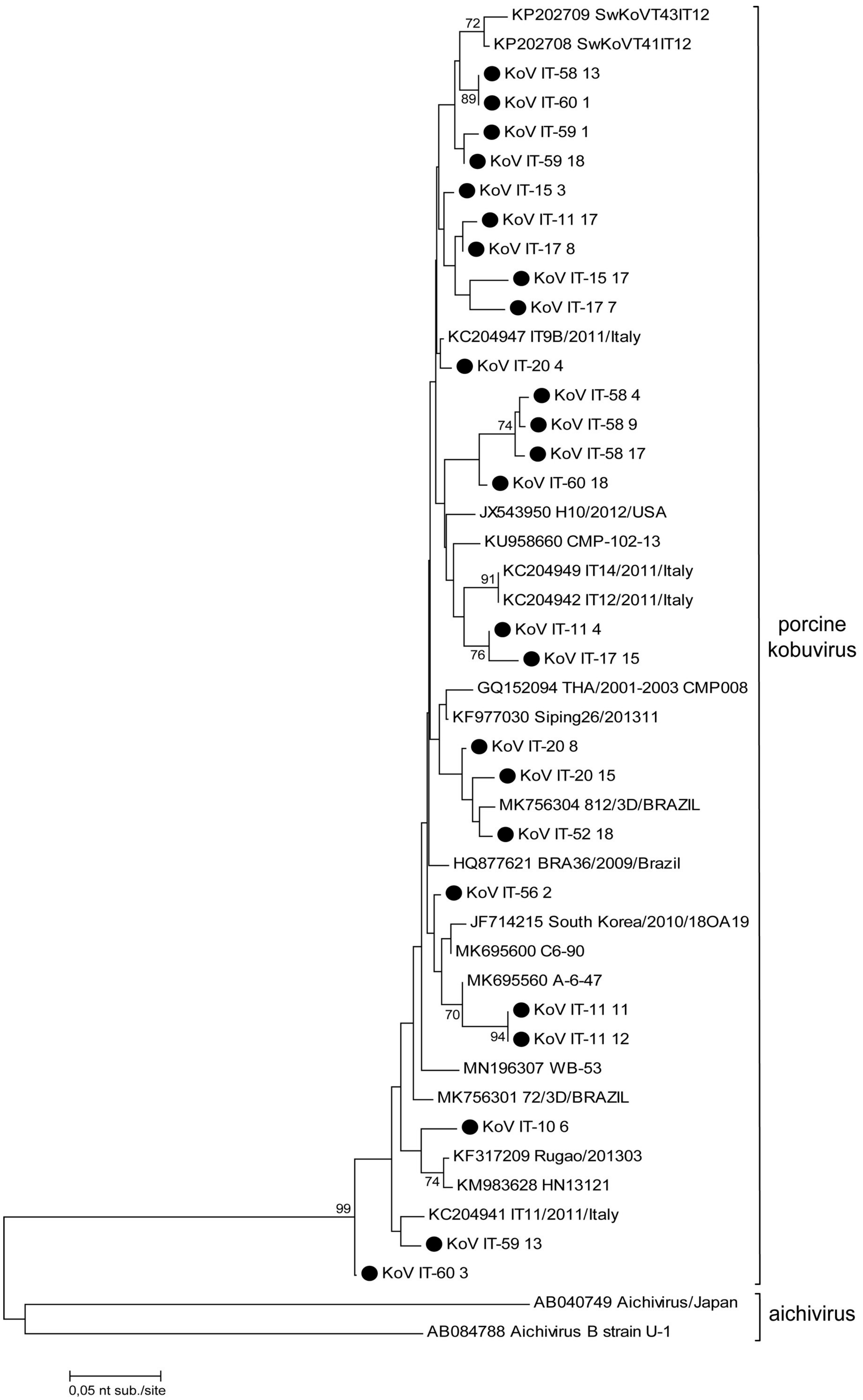
72
KP202709 SwKoVT43IT12
KP202708 SwKoVT41IT12
KoV IT-58 13
89
KoV IT-60 1
KoV IT-59 1
KoV IT-59 18
KoV IT-15 3
KoV IT-11 17
KoV IT-17 8
KoV IT-15 17
KoV IT-17 7
KC204947 IT9B/2011/Italy
KoV IT-20 4
KoV IT-58 4
74
KoV IT-58 9
KoV IT-58 17
KoV IT-60 18
JX543950 H10/2012/USA
KU958660 CMP-102-13
91
KC204949 IT14/2011/Italy
KC204942 IT12/2011/Italy
KoV IT-11 4
76
KoV IT-17 15
GQ152094 THA/2001-2003 CMP008
KF977030 Siping26/201311
KoV IT-20 8
KoV IT-20 15
MK756304 812/3D/BRAZIL
KoV IT-52 18
HQ877621 BRA36/2009/Brazil
KoV IT-56 2
JF714215 South Korea/2010/18OA19
MK695600 C6-90
MK695560 A-6-47
70
KoV IT-11 11
94
KoV IT-11 12
MN196307 WB-53
MK756301 72/3D/BRAZIL
KoV IT-10 6
KF317209 Rugao/201303
74
KM983628 HN13121
99
KC204941 IT11/2011/Italy
KoV IT-59 13
KoV IT-60 3
AB040749 Aichivirus/Japan
AB084788 Aichivirus B strain U-1
porcine kobuvirus
aichivirus
0,05 nt sub./site
