# Supplementary material for: Ten Years Later: Molecular Detection and Circulation of Enteric Viruses in Italian Pig Herds
Source: Transbound Emerg Dis. 2026 Oct 30;2026:4011873. doi: 10.1155/tbed/4011873 (PMC13721171; doi:10.1155/tbed/4011873)
Supplement: Supplementary file 2 [file TBED-2026-4011873-s002.docx]

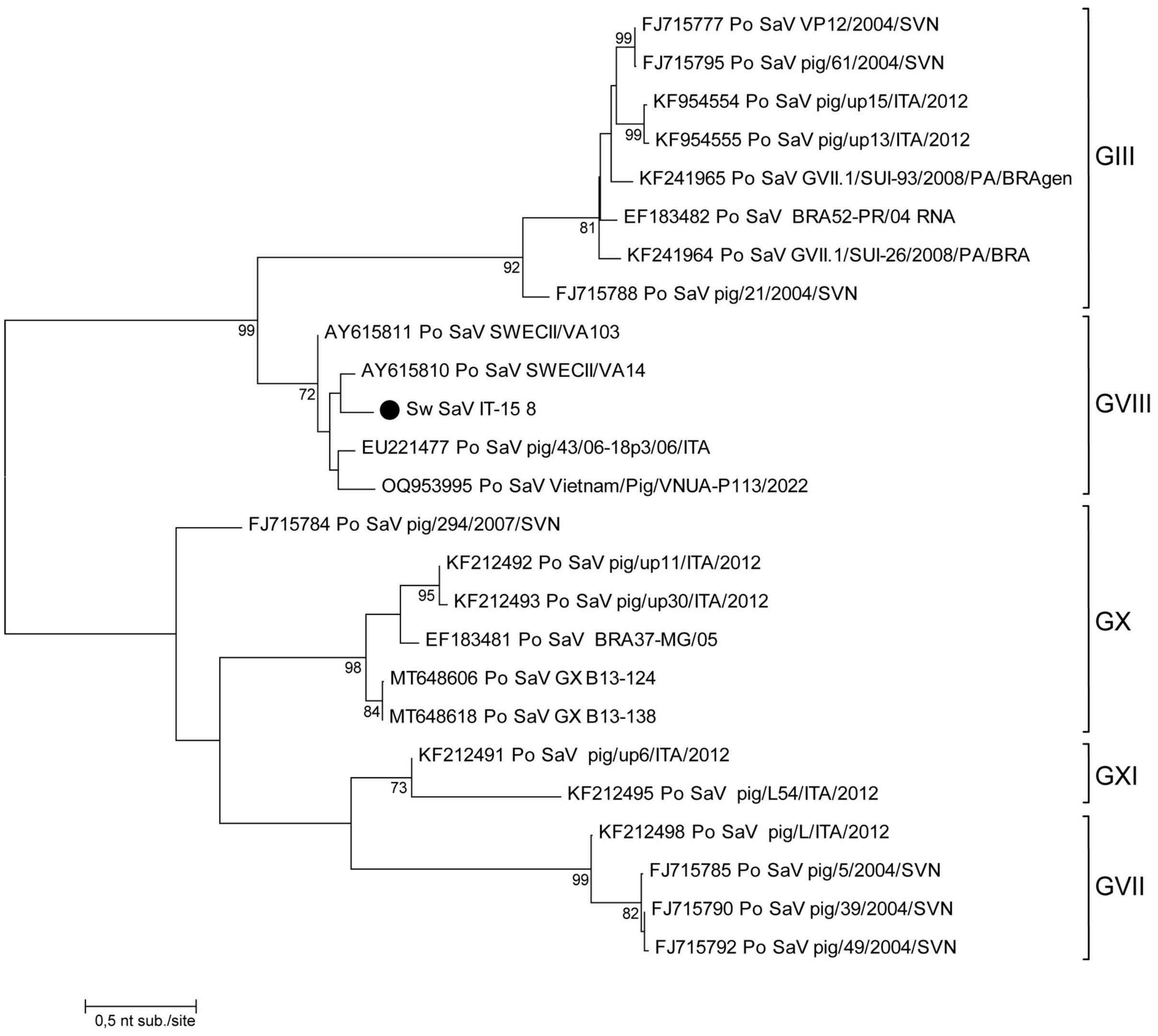

FJ715777 Po SaV VP12/2004/SVN
FJ715795 Po SaV pig/61/2004/SVN
KF954554 Po SaV pig/up15/ITA/2012
KF954555 Po SaV pig/up13/ITA/2012
KF241965 Po SaV GVII.1/SUI-93/2008/PA/BRAgen
EF183482 Po SaV BRA52-PR/04 RNA
KF241964 Po SaV GVII.1/SUI-26/2008/PA/BRA
FJ715788 Po SaV pig/21/2004/SVN
AY615811 Po SaV SWECII/VA103
AY615810 Po SaV SWECII/VA14
Sw SaV IT-15 8
EU221477 Po SaV pig/43/06-18p3/06/ITA
OQ953995 Po SaV Vietnam/Pig/VNUA-P113/2022
FJ715784 Po SaV pig/294/2007/SVN
KF212492 Po SaV pig/up11/ITA/2012
KF212493 Po SaV pig/up30/ITA/2012
EF183481 Po SaV BRA37-MG/05
MT648606 Po SaV GX B13-124
MT648618 Po SaV GX B13-138
KF212491 Po SaV pig/up6/ITA/2012
KF212495 Po SaV pig/L54/ITA/2012
KF212498 Po SaV pig/L/ITA/2012
FJ715785 Po SaV pig/5/2004/SVN
FJ715790 Po SaV pig/39/2004/SVN
FJ715792 Po SaV pig/49/2004/SVN
GIII
GVIII
GX
GXI
GVII
99
99
81
92
99
72
95
98
84
73
99
82
0,5 nt sub./site
