# Supplementary material for: Ten Years Later: Molecular Detection and Circulation of Enteric Viruses in Italian Pig Herds
Source: Transbound Emerg Dis. 2026 Oct 30;2026:4011873. doi: 10.1155/tbed/4011873 (PMC13721171; doi:10.1155/tbed/4011873)
Supplement: Supplementary file 3 [file TBED-2026-4011873-s003.docx]

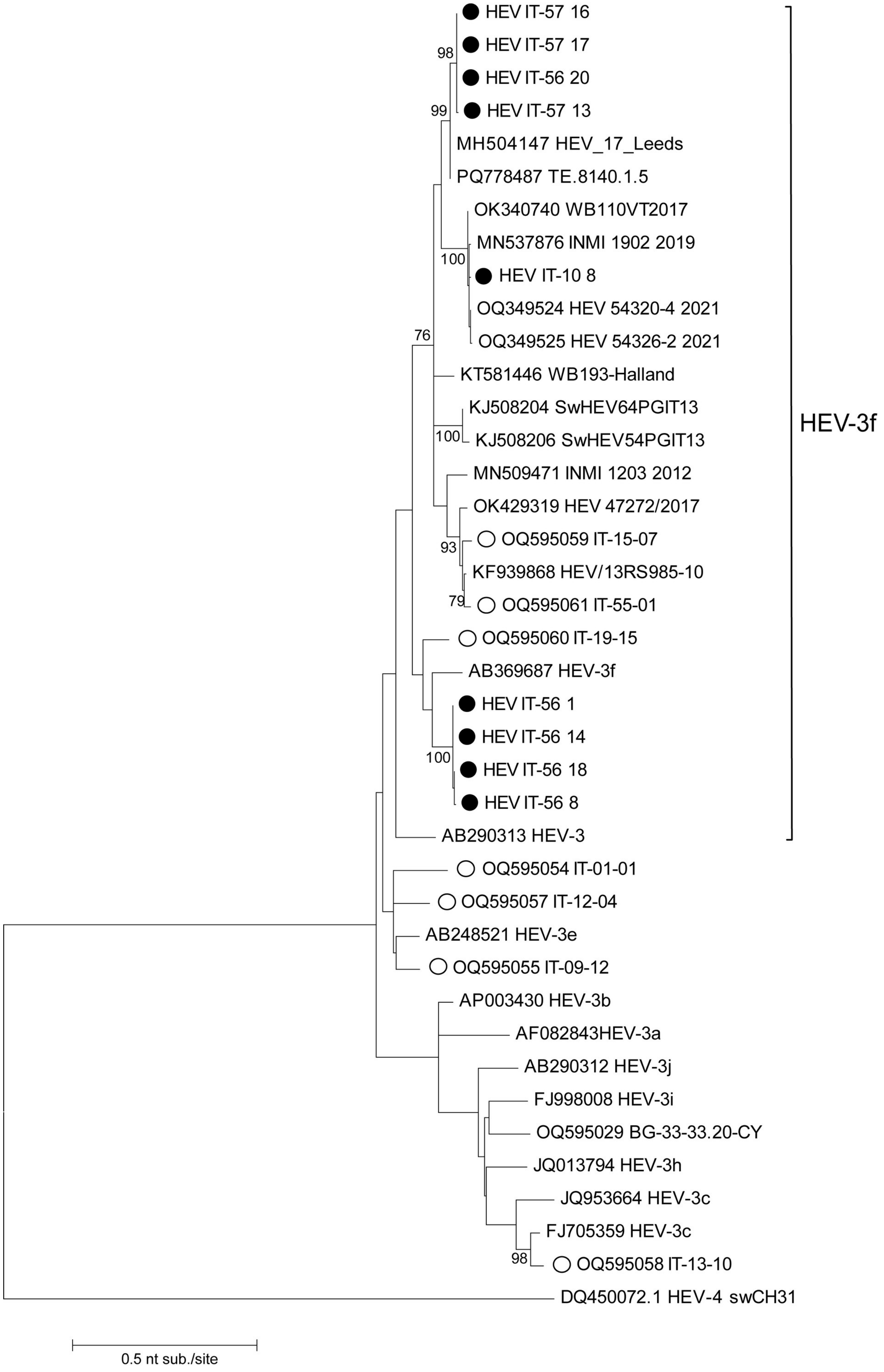
HEV IT-57 16
HEV IT-57 17
HEV IT-56 20
HEV IT-57 13
MH504147 HEV_17_Leeds
PQ778487 TE.8140.1.5
OK340740 WB110VT2017
MN537876 INMI 1902 2019
HEV IT-10 8
OQ349524 HEV 54320-4 2021
OQ349525 HEV 54326-2 2021
KT581446 WB193-Halland
KJ508204 SwHEV64PGIT13
KJ508206 SwHEV54PGIT13
MN509471 INMI 1203 2012
OK429319 HEV 47272/2017
OQ595059 IT-15-07
KF939868 HEV/13RS985-10
OQ595061 IT-55-01
OQ595060 IT-19-15
AB369687 HEV-3f
HEV IT-56 1
HEV IT-56 14
HEV IT-56 18
HEV IT-56 8
AB290313 HEV-3
HEV-3f
OQ595054 IT-01-01
OQ595057 IT-12-04
AB248521 HEV-3e
OQ595055 IT-09-12
AP003430 HEV-3b
AF082843HEV-3a
AB290312 HEV-3j
FJ998008 HEV-3i
OQ595029 BG-33-33.20-CY
JQ013794 HEV-3h
JQ953664 HEV-3c
FJ705359 HEV-3c
OQ595058 IT-13-10
DQ450072.1 HEV-4 swCH31
98
99
100
76
100
93
79
100
98
0.5 nt sub./site
