# Supplementary material for: Ten Years Later: Molecular Detection and Circulation of Enteric Viruses in Italian Pig Herds
Source: Transbound Emerg Dis. 2026 Oct 30;2026:4011873. doi: 10.1155/tbed/4011873 (PMC13721171; doi:10.1155/tbed/4011873)
Supplement: Supplementary file 4 [file TBED-2026-4011873-s004.docx]

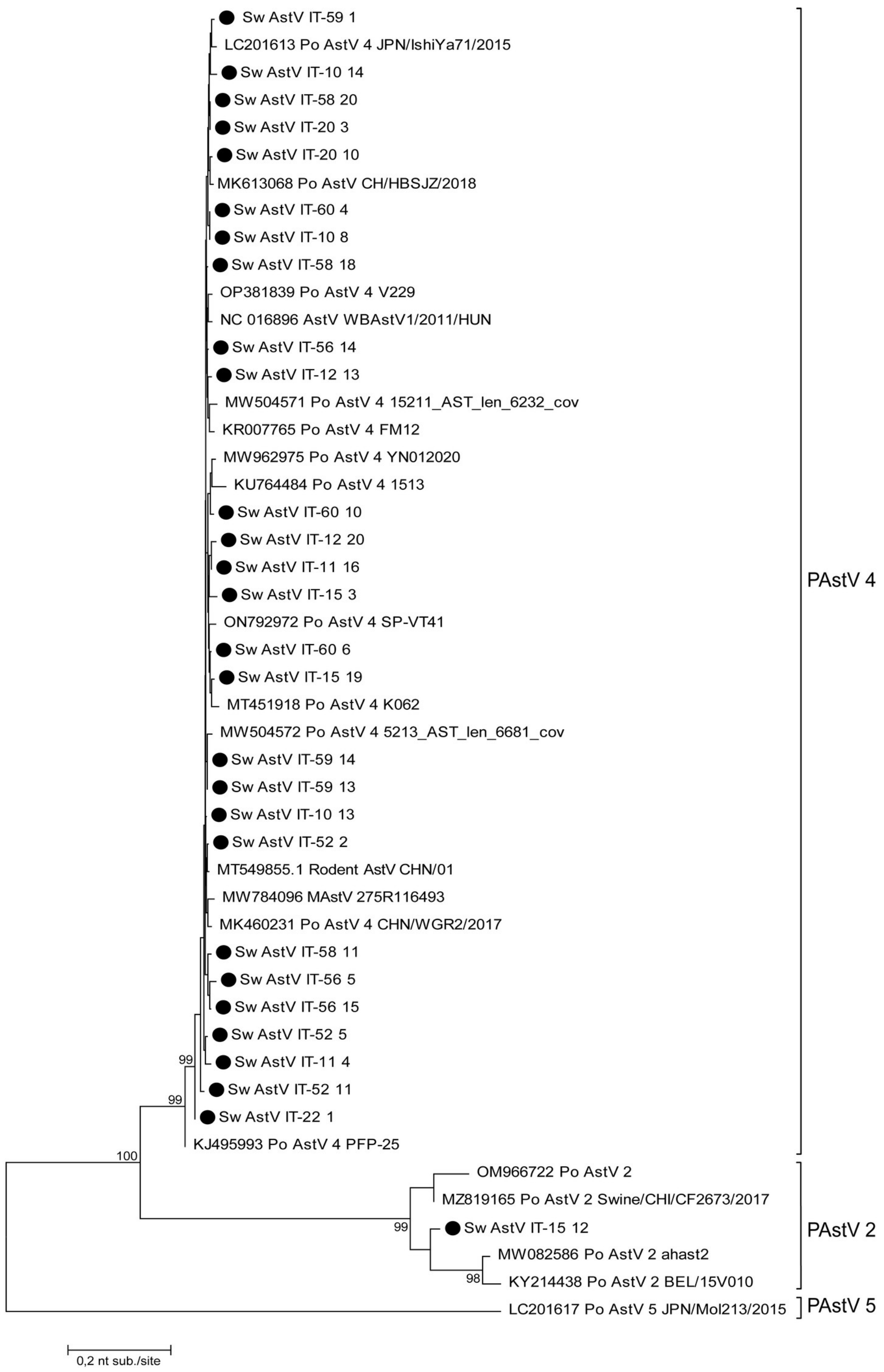

Sw AstV IT-59 1
LC201613 Po AstV 4 JPN/IshiYa71/2015
Sw AstV IT-10 14
Sw AstV IT-58 20
Sw AstV IT-20 3
Sw AstV IT-20 10
MK613068 Po AstV CH/HBSJZ/2018
Sw AstV IT-60 4
Sw AstV IT-10 8
Sw AstV IT-58 18
OP381839 Po AstV 4 V229
NC 016896 AstV WBAstV1/2011/HUN
Sw AstV IT-56 14
Sw AstV IT-12 13
MW504571 Po AstV 4 15211_AST_len_6232_cov
KR007765 Po AstV 4 FM12
MW962975 Po AstV 4 YN012020
KU764484 Po AstV 4 1513
Sw AstV IT-60 10
Sw AstV IT-12 20
Sw AstV IT-11 16
Sw AstV IT-15 3
ON792972 Po AstV 4 SP-VT41
Sw AstV IT-60 6
Sw AstV IT-15 19
MT451918 Po AstV 4 K062
MW504572 Po AstV 4 5213_AST_len_6681_cov
Sw AstV IT-59 14
Sw AstV IT-59 13
Sw AstV IT-10 13
Sw AstV IT-52 2
MT549855.1 Rodent AstV CHN/01
MW784096 MAstV 275R116493
MK460231 Po AstV 4 CHN/WGR2/2017
Sw AstV IT-58 11
Sw AstV IT-56 5
Sw AstV IT-56 15
Sw AstV IT-52 5
Sw AstV IT-11 4
Sw AstV IT-52 11
Sw AstV IT-22 1
KJ495993 Po AstV 4 PFP-25
99
99
100
PAstV 4
OM966722 Po AstV 2
MZ819165 Po AstV 2 Swine/CHI/CF2673/2017
Sw AstV IT-15 12
MW082586 Po AstV 2 ahast2
KY214438 Po AstV 2 BEL/15V010
99
98
PAstV 2
LC201617 Po AstV 5 JPN/Mol213/2015
PAstV 5
0,2 nt sub./site
