# Supplementary material for: Ten Years Later: Molecular Detection and Circulation of Enteric Viruses in Italian Pig Herds
Source: Transbound Emerg Dis. 2026 Oct 30;2026:4011873. doi: 10.1155/tbed/4011873 (PMC13721171; doi:10.1155/tbed/4011873)
Supplement: Supplementary file 5 [file TBED-2026-4011873-s005.docx]

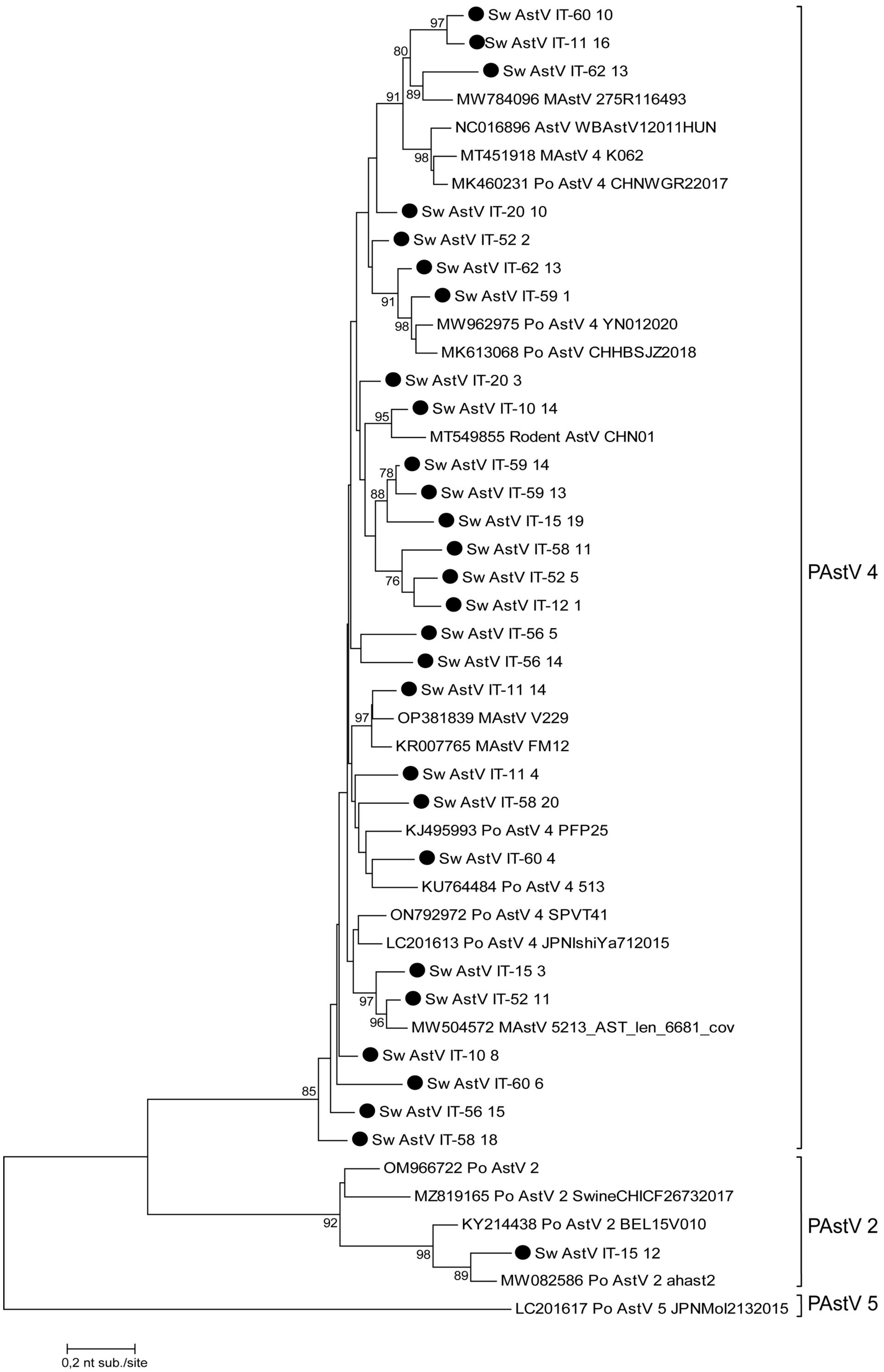
Sw AstV IT-60 10
Sw AstV IT-11 16
Sw AstV IT-62 13
MW784096 MAstV 275R116493
NC016896 AstV WBAstV12011HUN
MT451918 MAstV 4 K062
MK460231 Po AstV 4 CHNWGR22017
Sw AstV IT-20 10
Sw AstV IT-52 2
Sw AstV IT-62 13
Sw AstV IT-59 1
MW962975 Po AstV 4 YN012020
MK613068 Po AstV CHHBSJZ2018
Sw AstV IT-20 3
Sw AstV IT-10 14
MT549855 Rodent AstV CHN01
Sw AstV IT-59 14
Sw AstV IT-59 13
Sw AstV IT-15 19
Sw AstV IT-58 11
Sw AstV IT-52 5
Sw AstV IT-12 1
Sw AstV IT-56 5
Sw AstV IT-56 14
Sw AstV IT-11 14
OP381839 MAstV V229
KR007765 MAstV FM12
Sw AstV IT-11 4
Sw AstV IT-58 20
KJ495993 Po AstV 4 PFP25
Sw AstV IT-60 4
KU764484 Po AstV 4 513
ON792972 Po AstV 4 SPVT41
LC201613 Po AstV 4 JPNIshiYa712015
Sw AstV IT-15 3
Sw AstV IT-52 11
MW504572 MAstV 5213_AST_len_6681_cov
Sw AstV IT-10 8
Sw AstV IT-60 6
Sw AstV IT-56 15
Sw AstV IT-58 18
OM966722 Po AstV 2
MZ819165 Po AstV 2 SwineCHICF26732017
KY214438 Po AstV 2 BEL15V010
Sw AstV IT-15 12
MW082586 Po AstV 2 ahast2
LC201617 Po AstV 5 JPNMol2132015
PAstV 4
PAstV 2
PAstV 5
0,2 nt sub./site
