# Supplementary material for: Ten Years Later: Molecular Detection and Circulation of Enteric Viruses in Italian Pig Herds
Source: Transbound Emerg Dis. 2026 Oct 30;2026:4011873. doi: 10.1155/tbed/4011873 (PMC13721171; doi:10.1155/tbed/4011873)
Supplement: Supplementary file 6 [file TBED-2026-4011873-s006.docx]

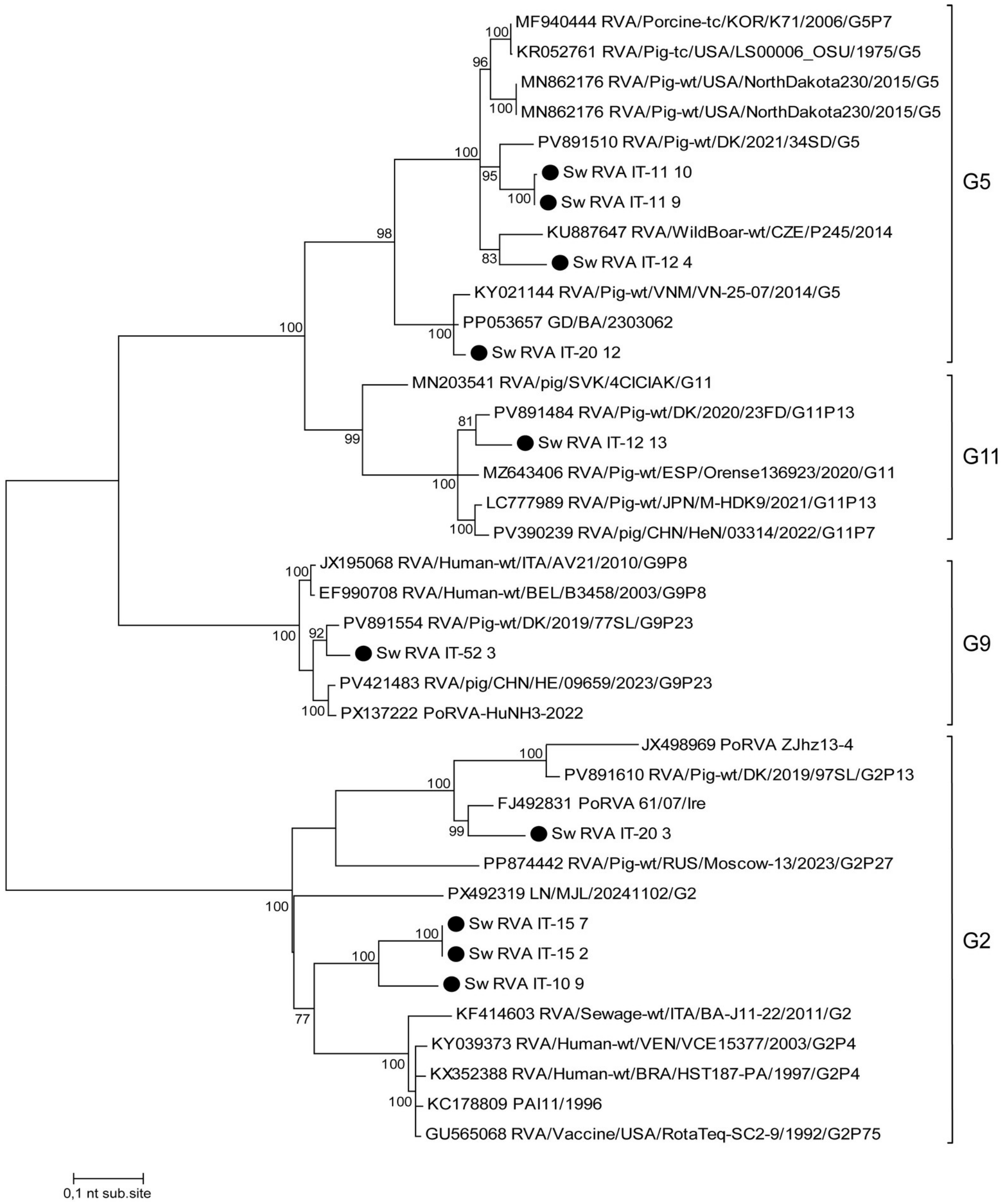
MF940444 RVA/Porcine-tc/KOR/K71/2006/G5P7
KR052761 RVA/Pig-tc/USA/LS00006_OSU/1975/G5
MN862176 RVA/Pig-wt/USA/NorthDakota230/2015/G5
MN862176 RVA/Pig-wt/USA/NorthDakota230/2015/G5
PV891510 RVA/Pig-wt/DK/2021/34SD/G5
Sw RVA IT-11 10
Sw RVA IT-11 9
KU887647 RVA/WildBoar-wt/CZE/P245/2014
Sw RVA IT-12 4
KY021144 RVA/Pig-wt/VNM/VN-25-07/2014/G5
PP053657 GD/BA/2303062
Sw RVA IT-20 12
MN203541 RVA/pig/SVK/4ClClAK/G11
PV891484 RVA/Pig-wt/DK/2020/23FD/G11P13
Sw RVA IT-12 13
MZ643406 RVA/Pig-wt/ESP/Orense136923/2020/G11
LC777989 RVA/Pig-wt/JPN/M-HDK9/2021/G11P13
PV390239 RVA/pig/CHN/HeN/03314/2022/G11P7
JX195068 RVA/Human-wt/ITA/AV21/2010/G9P8
EF990708 RVA/Human-wt/BEL/B3458/2003/G9P8
PV891554 RVA/Pig-wt/DK/2019/77SL/G9P23
Sw RVA IT-52 3
PV421483 RVA/pig/CHN/HE/09659/2023/G9P23
PX137222 PoRVA-HuNH3-2022
JX498969 PoRVA ZJhz13-4
PV891610 RVA/Pig-wt/DK/2019/97SL/G2P13
FJ492831 PoRVA 61/07/Ire
Sw RVA IT-20 3
PP874442 RVA/Pig-wt/RUS/Moscow-13/2023/G2P27
PX492319 LN/MJL/20241102/G2
Sw RVA IT-15 7
Sw RVA IT-15 2
Sw RVA IT-10 9
KF414603 RVA/Sewage-wt/ITA/BA-J11-22/2011/G2
KY039373 RVA/Human-wt/VEN/VCE15377/2003/G2P4
KX352388 RVA/Human-wt/BRA/HST187-PA/1997/G2P4
KC178809 PAI11/1996
GU565068 RVA/Vaccine/USA/RotaTeq-SC2-9/1992/G2P75
G5
G11
G9
G2
100
96
100
100
95
100
83
98
100
100
81
99
100
100
100
100
92
100
100
100
99
100
100
100
77
100
100
0,1 nt sub.site
