# Supplementary material for: Ten Years Later: Molecular Detection and Circulation of Enteric Viruses in Italian Pig Herds
Source: Transbound Emerg Dis. 2026 Oct 30;2026:4011873. doi: 10.1155/tbed/4011873 (PMC13721171; doi:10.1155/tbed/4011873)
Supplement: Supplementary file 7 [file TBED-2026-4011873-s007.docx]

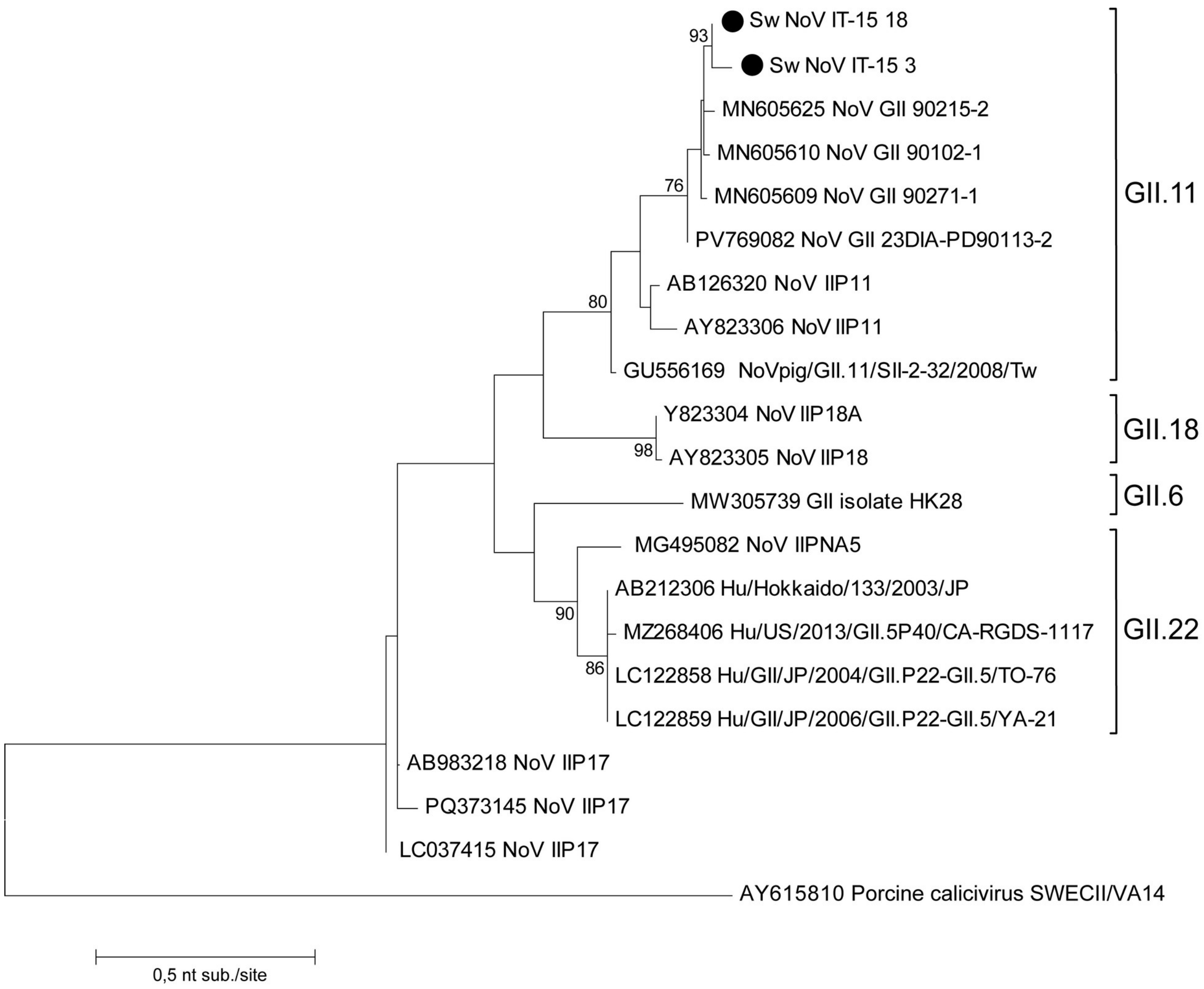

Sw NoV IT-15 18
Sw NoV IT-15 3
MN605625 NoV GII 90215-2
MN605610 NoV GII 90102-1
MN605609 NoV GII 90271-1
PV769082 NoV GII 23DIA-PD90113-2
AB126320 NoV IIP11
AY823306 NoV IIP11
GU556169 NoVpig/GII.11/SII-2-32/2008/Tw
Y823304 NoV IIP18A
AY823305 NoV IIP18
MW305739 GII isolate HK28
MG495082 NoV IIPNA5
AB212306 Hu/Hokkaido/133/2003/JP
MZ268406 Hu/US/2013/GII.5P40/CA-RGDS-1117
LC122858 Hu/GII/JP/2004/GII.P22-GII.5/TO-76
LC122859 Hu/GII/JP/2006/GII.P22-GII.5/YA-21
AB983218 NoV IIP17
PQ373145 NoV IIP17
LC037415 NoV IIP17
AY615810 Porcine calicivirus SWECII/VA14
93
76
80
98
90
86
GII.11
GII.18
GII.6
GII.22
0,5 nt sub./site
